# Conjugated nanoliposome with the HER2/neu-derived peptide GP2 as an effective vaccine against breast cancer in mice xenograft model

**DOI:** 10.1371/journal.pone.0185099

**Published:** 2017-10-18

**Authors:** Atefeh Razazan, Javad Behravan, Atefeh Arab, Nastaran Barati, Leila Arabi, Zahra Gholizadeh, Mahdi Hatamipour, Amin Reza Nikpoor, Amir Abbas Momtazi-Borojeni, Fatemeh Mosaffa, Mohamad Hosein Ghahremani, Mahmoud Reza Jaafari

**Affiliations:** 1 Department of Molecular Medicine, School of Advanced Technologies in Medicine, Tehran University of Medical Sciences, Tehran Iran; 2 Nanotechnology Research Center, Mashhad University of Medical Sciences, Mashhad, Iran; 3 Biotechnology Research Center, Mashhad University of Medical Sciences, Mashhad, Iran; 4 School of Pharmacy, Mashhad University of Medical Sciences, Mashhad, Iran; 5 Department of Immunology, School of Medicine, Mashhad University of Medical Sciences, Mashhad, Iran; 6 Student Research Committee, Department of Medical Biotechnology, Faculty of Medicine, Mashhad University of Medical Sciences, Mashhad, Iran; Universita degli Studi di Catania, ITALY

## Abstract

One of the challenging issues in vaccine development is peptide and adjuvant delivery into target cells. In this study, we developed a vaccine and therapeutic delivery system to increase cytotoxic T lymphocyte (CTL) response against a breast cancer model overexpressing HER2/neu.

Gp2, a HER2/neu-derived peptide, was conjugated to Maleimide-mPEG_2000-_DSPE micelles and post inserted into liposomes composed of DMPC, DMPG phospholipids, and fusogenic lipid dioleoylphosphatidylethanolamine (DOPE) containing monophosphoryl lipid A (MPL) adjuvant (DMPC-DMPG-DOPE-MPL-Gp2). BALB/c mice were immunized with different formulations and the immune response was evaluated *in vitro* and *in vivo*. ELISpot and intracellular cytokine analysis by flow cytometry showed that the mice vaccinated with Lip-DOPE-MPL-GP2 incited the highest number of IFN-γ+ in CD8+ cells and CTL response. The immunization led to lower tumor sizes and longer survival time compared to the other groups of mice immunized and treated with the Lip-DOPE-MPL-GP2 formulation in both prophylactic and therapeutic experiments. These results showed that co-formulation of DOPE and MPL conjugated with GP2 peptide not only induces high antitumor immunity but also enhances therapeutic efficacy in TUBO mice model. Lip-DOPE-MPL-GP2 formulation could be a promising vaccine and a therapeutic delivery system against HER2 positive cancers and merits further investigation.

## Introduction

Human epidermal growth factor-like receptor (HER2/neu) is an oncogene that belongs to the transmembrane receptor family with 100 folds higher expression in tumor cells than normal tissues [1]. HER2/neu as an immunogenic protein elicits both humoral and cellular immune responses. This oncogene is an important biomarker and the goal of therapy for almost 30% of all breast cancers[2].

Immunotherapy is a treatment that uses certain parts of a person’s immune system to attack cancer cells specifically[3]. Many Tumor-Associated Antigens (TAAs) can be recognized by the immune system [4]. Cancer vaccines belong to a class of substances known as biological response modifiers that work by stimulating or restoring the immune system’s ability to fight infections and disease. There are two broad types of cancer vaccines: prophylactic vaccines, which are intended to prevent cancer from developing in healthy people, and the second group, therapeutic vaccines, are intended to treat existing cancers by strengthening the body's natural immune response [5].

Peptides used in vaccines are antigenic epitopes derived from TAAs, which can stimulate immune regulators including antibodies, helper T cells, and cytotoxic T lymphocytes [6].Advantages of peptide vaccines are stability, simple component, and low microbial infection [7].

GP2 is an immunogenic peptide that is recognized by the endogenous immune system [8].GP2 with nine amino acids (654–662: IISAVVGIL) is a MHC class I peptide which is derived from HER2 protein's transmembrane domain. GP2 is an efficient peptide for producing a multi-epitope vaccine [9, 10]. The results indicated that GP2 peptide can induce a CTL response similar to E75 and it is equally or more immunogenic [11]. Phase I clinical trial demonstrated that GP2 peptide is safe and capable of stimulating a her2-specific immune response [12]. Because GP2 peptide has a strong potential immunogenicity, it is an appropriate candidate for peptide vaccine trials [11].

In an immunization formulation, adjuvant components are used to obtain higher stimulation and elongation time of the special immune system against antigens [13]. Toll like receptor (TLR) agonists are novel immunostimulatory adjuvants that can be used for human vaccines[14]. Monophosphoryl lipid A (MPL), a TLR4 agonist, is a chemically detoxified derivative of the parent lipopolysaccharide (LPS) from *Salmonella minnesota* R595 strain. MPL can provoke dendritic cells to secrete inflammatory cytokines such as INF-γ, IL-12, and IL-6 and also activate the T cell immune response [15–17]. MLP has been used as a potent adjuvant for designing vaccines against some important human diseases such as malaria, HIV-1, meningococcal type B disease, breast cancer, prostate cancer, and colon cancer [18]. Therefore, co-delivery of antigen and adjuvant is essential for impelling an adequate immune response [19].

Liposomes have been extensively studied as carriers for delivering antigens and adjuvants to antigen-presenting cells (APCs). Efficient delivery of TAAs to the major histocompatibility complex class I presentation pathway in APCs will substantially contribute to establishing more effective cancer immunotherapy. The first use of a peptide to raise the immune response to loaded liposomes was reported by Allison [20].

In the current study, to increase carrier delivery efficiency we used the helper-fusogenic lipids such as DOPE. DOPE transfers from a bilayer structure to hexagonal phase as an endosome acidifies and delivers the antigens to the cytosol of APCs [21, 22].

There are two ways for liposome antigen incorporation: covalent linking and encapsulation. Reportedly, linking of antigen to liposome has higher efficiency in cell mediated immunity response compared to antigen encapsulation [23]. In our previous study, P5 peptide conjugated toMaleimide-PEG_2000_-DSPE with MPL adjuvant induced strong CTL response that reduced tumor growth with prolonged survival time in the TUBO tumor mice model [24].

In this study, we aimed at developing a liposomal vaccine composed of DMPC- DMPG- Chol- DOPE containing MPL with Gp2 peptide conjugated to the surface of liposomes to increase the CTL response and cellular immunity in the BALB/c mice model of TUBO xenograft cancer.

## Materials and methods

### Animals

Four to six week-old female BALB/c mice were purchased from the Pasteur Institute (Tehran-Iran). All procedures involving animal and the proposal were approved by the Institutional Ethical Committee and Research Advisory Committee of Mashhad University of Medical Sciences in accordance with the Animal Welfare Guideline (Education Office, dated Feb.28.2014; proposal code 98623). Animals were kept in cages and provided with food and water *ad libitum*. All mice received humane care in compliance with the institutional guideline.

### Cell lines

TUBO, a cloned cell line that overexpressed the rHER2/neu protein, was kindly provided by Dr. Pier-Luigi Lollini (Department of Clinical and Biological Sciences, University of Turin, Orbassano, Italy) and was cultured in Dulbecco's Modified Eagle's Medium (DMEM) and supplemented with 20% fetal bovine serum (FBS). CT26, a murine colon carcinoma cell line was purchased from Pasture Institute (Tehran-Iran) and cultured in RPMI-1640 medium supplemented with 10% FBS. CT26 cells (rHER2/neu negative) were used as negative control.

### Peptide and chemicals

GP2 (Ac-CGGGIISAVVGIL) with 99.95% purity and molecular weight of 1.2 KD was synthesized by China Peptides Co. Ltd (Shanghai, China). DMPC (Dimyristoylphosphatidylcoline), DMPG (Dimyristoylphosphoglycerol), DOPE (dioleoylphosphatidylethanolamine) and Distearoylphsphoethanolamin-N- [Maleimide (polyethylene glycol)-2000] (Maleimide-PEG_2000_-DSPE) were purchased from Avanti Polar lipid (Alabaster, USA) and cholesterol and Monophosphoryl lipid A (MPL) were purchased from Sigma Aldrich (Steinheim, Germany). Cytofix/Cytoperm ™ Plus, PMA/ionomycin cocktail, anti-CD8a-PE-cy5, anti- CD4-PE-cy5, anti-INF-γ-FITC, and anti-IL-4 PE antibodies were purchased from BD Biosciences (SanDiego, USA).

### Conjugation of GP2 peptide to PEG_2000_-DSPE

GP2 peptide (dissolved in dimethyl sulfoxide, DMSO) and maleimide-PEG_2000_-DSPE (dissolved in chloroform), weremixed atmolar ratios of 1.2:1 in a sterile glass tube at 37°C for 48h. The mixture was dried using a rotary evaporator and freeze-dryer, which was followed by hydration with sterile water and bath-sonication for 5 min at 25°C.

Thin layer chromatography (TLC) and SDS-PAGE were used to confirm the binding between the thiol group of peptide cysteine residue and the pyrrole group of maleimide. A TLC plate (silica gel 60 F25A, Merck, USA) was applied in a TLC chamber containing mobile phase composed of chloroform, methanol, and water at 90:18:2 (v/v) ratios, then the TLC chamber was saturated with iodine vapor to stain the TLC plate.

The SDS-PAGE consisted of running gel (16% (w/v) acrylamide / 6M urea), stacking gel (4% (w/v) acrylamide), and spacer gel (10% (w/v) acrylamide);the gel thickness was 0.7 mm. The anode buffer was 0.1 M Tris, pH 8.9 and cathode buffer was 0.1 M Tris, 0.1 M Tricine, 0.1% SDS, pH 8.25. Electrophoresis was carried out with an initial voltage of 30 V, which was increased to 300 V at the end of the run then stained with silver nitrate for visualization[25].

In addition, HPLC method was used to verify linking of peptide to maleimide by determining the fraction of free peptide. KNAUER smart line HPLC (Berlin, Germany) was equipped with a Nucleosil C18, 5μm, 150 × 4.6mm, and 100 A° column (KNAUER) and a UV detector (KNAUER S2600) set at 220 nm. The flow rate was set to 1 ml/min and the mobile phases employed were A (water + 0.1% TFA) and B (acetonitrile+0.1%TFA). The gradient elution program started with 100% A and was increased to 20% B in 2 min and 80% A in 2 min.

### Preparation of nanoliposomes

Liposomes (Lip-DOPE) composed of DMPC:DMPG:Chol:DOPE at molar ratios 30:4:6:10 and 0.25 mg/ml MPL with lipid concentration of 40 mM were prepared by dissolving the lipids in chloroform. Rotary evaporation at 30°C (Heidolph, Germany) and freeze drying (VD-800f, Taitech, Japan) were used to form the thin lipid film. HEPES buffer, 5% dextrose (10 mM, pH 7.2) were used for thin film hydration followed by vortexing for 10 min until the thin film was dissolved with HEPS-dextrose 5%(HEPS-dext 5%) completely. The vesicles were extruded through 800 nm, 400 nm, and 100nm polycarbonate membranes using an extruder (Avestin, Canada). To prepare Lip-MPL-GP2, GP2-PEG_2000_-DSPE micelles were post inserted into the liposomes containing MPL at 45°C with 250 RPM (Innova 4080 Incubator shaker) shaking for 4h.

### Characterization of nanoliposomes

GP2 peptide content in liposomal formulations was determined using an HPLC method. The amount of lipids was determined by the Bartlett phosphate assay method[26]. In order to disrupt liposomes, 1.5% (v/v) C_12_E_10_ detergent was added to the formulations then the MPL content was assayed by the LAL chromogenic endpoint assay (QCL-1000,Lonza,Walkersville,MD)[27]. Vesicle size, zeta potential, and polydispersity index of liposomes were determined by dynamic light scattering (Malvern Instruments, Malvern, UK).

### Immunization of BALB/c mice

BALB/c mice were divided into eight groups (eight mice per group). Each liposomal formulation (5 μmol per mice containing 10 μg of GP2 peptide) was injected subcutaneously (SC) three times in two-week intervals. The free GP2 peptide 10 μg per mice and HEPS-dextrose 5% were used as control. Two weeks after the last booster, three mice per group were anesthetized with injection of 0.1 ml of the ketamine-xylazine solution per 10 g of body weight intraperitoneally (ketamine (100 mg/kg) and xylazine (10 mg/kg))[28]. 20–25 min elapsed before euthanasia for each mouse; they were scarified by cervical dislocation, then splenocytes were aseptically removed and the cellular immune responses were evaluated. None of the animals died before meeting criteria for euthanasia.

### Enzyme-linked immunospot (ELISpot) assay

The ELISpot assay was used for the evaluation of INF-γ and IL-4 expression in the splenocytes in response to the peptide. Mouse ELISpot kit from U-cyten (Utrecht, Netherlands) was used according to the manufacturer’s instruction. Briefly, ELISpot 96-well plates were coated with anti-IL4 and anti-INF-γ antibodies. After overnight incubation at 4°C, splenocytes isolated from sacrificed mice were cultured in triplicate wells in pre-coated plates with medium containing GP2 peptide (10 μg/ml) and incubated for 24 h at 37°C. After spots appeared, counting was performed with Kodak 1D image analysis software (version 3.5, Eastman Kodak, Rochester, New York).

### *In vitro* CTL assay

Two weeks after the final vaccination, splenocytes were isolated from mice (three mice per group). Re-stimulation was performed with the GP2 peptide (10 μg/ml) and recombinant IL-2 (20 U/ml) for five days. Then TUBO cells (target cells) were incubated with 12.5 μm calcein AM (Calcein-AM, Invitrogen, USA) at 37°C for one hour in the dark[29]. Tritonx-100 2% and culture medium were added to the maximum and minimum release wells, respectively. Fluorescence intensity was measured at excitation of 485 nm and emission of 538 nm using a fluorescent plate reader (FLX 800, BioTek Instruments Inc. USA). The percentage of specific lysis was calculated by the following formula: (release by CTLs-minimum release by targets)/ (maximum release by targets-minimum release by targets). To show the specificity of cytotoxic activity, non-expressing rHER2/neuCT26 cells were used as negative control.

### Intracellular cytokine assay

To measure intracellular cytokines,10^6^ cells/ml of splenocytes and 10^6^ cells/ml of lymph nodes in a medium containing Golgiplug™ 1μl/ml (BD Biosciences, California, USA) were stimulated with 2 μl/ml PMA/ionomycin cocktail at 37°C for 4h. Then, 10^5^ splenocytes were washed with 2% FCS in PBS and stained with 1μl anti-CD8a-PE-cy5 and 1μl CD4-PE-cy5 antibodies (BD Biosciences) in separate tubes at 4°C for 30 min. For intracellular staining, cells were washed with staining buffer and fixed with cytofix/cytoperm™ solution then incubated for 20 min. After washing the fixed cells with the Perm/Wash™ solution, the cells were incubated with 1μl anti-INF-γ-FITC antibody. CD4 cells were separately stained with1μl anti-IL4-PE antibody at 4°C for 30 min. Finally, cells were washed with the Perm/Wash™ solution and suspended in staining buffer. Stained cells were analyzed using FACS Calibur™ (BD Biosciences, San Jose, USA).

### RNA extraction and real-time quantitative reverse transcription PCR

Real-time Reverse Transcription-PCR (RT-PCR) assay was employed to evaluate mRNA expression of INF-γ and IL-4 cytokines in splenocytes isolated from spleen immunized mice. Total RNA was extracted from homogenized spleen tissue using High Pure RNA Tissue Kit (Roche, Germany) as instructed by the manufacturer. The extracted RNA was quantified using a Nano Drop spectrophotometer (ND-1000) and samples were stored at -80°C until use.

The total RNA (100 ng) was used in real time RT-PCR using one-step SYBR Green real time RT-PCR kit according to manufacturer's instructions (Invitrogen, California, USA). The Applied Biosystems StepOne Real-time PCR System (Life Technologies Corporation, Carlsbad, CA) was used for one-step real time RT-PCR amplification and SYBR Green fluorescence detection. Briefly, the RT step performed at 50°C for five min followed by real time PCR reaction, involved an initial denaturation step at 95°C for two min and 40 cycles of 95°C for 15 sec and 60°C for one min.

Three pairs of primers were separately used: two pairs to amplify the INF-γ (F: GCTCTGAGACAATGAACGCT and R: AAAGAGATAATCTGGCTCTGC), IL-4 genes (F: TCGGCATTTTGAACGAGGTC and R: GAAAAGCCCGAAAGAGTCTC and the other pair for the endogenous control gene β-actin (F: TGACCGGCTTGTATGCTATC and R: CAGTGTGAGCCAGGATATAG)[25, 30]. A negative control was included in each run to access specificity of primers and possible contamination. The possibility of nonspecific amplification or primer-dimmer formation was checked using melt curve analysis.

The comparative C_T_ (threshold cycle) method was used to evaluate fold changes of mRNA levels in the immunized group relative to the control group. The fluorescence C_T_ was calculated using Step One system software. The mRNA levels were normalized to the endogenous reference gene β-actin (ΔC_T_) and then relative to a control group (ΔΔCT), subsequently fold change was expressed as “log2 [2^(-ΔΔCT)^]”. The average was calculated from three runs per sample.

### *In vivo* prophylactic studies

Fourteen days after the last vaccination, 5×10 ^5^ TUBO cells in 50 μl PBS buffer were injected SC in the right flank of immunized mice (five mice per group). Mice were monitored every day. Three orthogonal diameters (a,b,c) were measured with a digital caliper. The tumor volume was calculated according to the formulation [(height × width × length) × 0.5]. The equation of the line obtained by exponential regression of the tumor growth curve was used for TTE (time to reach the end point) and the difference between the median TTE of the treatment group (T) and the median TTE of the control group (C) was used to calculate %TGD (the percent of tumor growth delay)(%TGD = [(T-C)/C] × 100])for each mouse [31, 32]. For ethical considerations, mice were sacrificed if the following conditions were observed: the tumor volume was greater than 1000 mm^3^, the body weight loss was over 15% of initial weight, or the mice became sick and unable to feed.

### *In vivo* therapeutic studies

5×10 ^5^ TUBO cells in 50μl PBS buffer were injected in the right flank of 4–6 week old female BALB/c mice. Two weeks after tumor inoculation, different liposomal formulations (100μl/mice) were injected SC three times at 2-week intervals. The free GP2 peptide (10μg per mouse) and HEPES dextrose 5% were used as control groups. Mice were monitored every day and the tumor volume was calculated as mentioned above.

### Statistical analysis

Descriptive statistics, One- way ANOVA, Tukey’s test, independent t-test, and log-rank test for survival analysis were used to assess the significance of the difference among various formulations (Graph Pad Prism Software, version 6, San Diego, CA). The *P*-value <0.05 (*p*<0.05) was considered to be statistically significant.

## Results

### Conjugation between peptide and maleimide-PEG_2000_-DSPE

**Fig 1:**
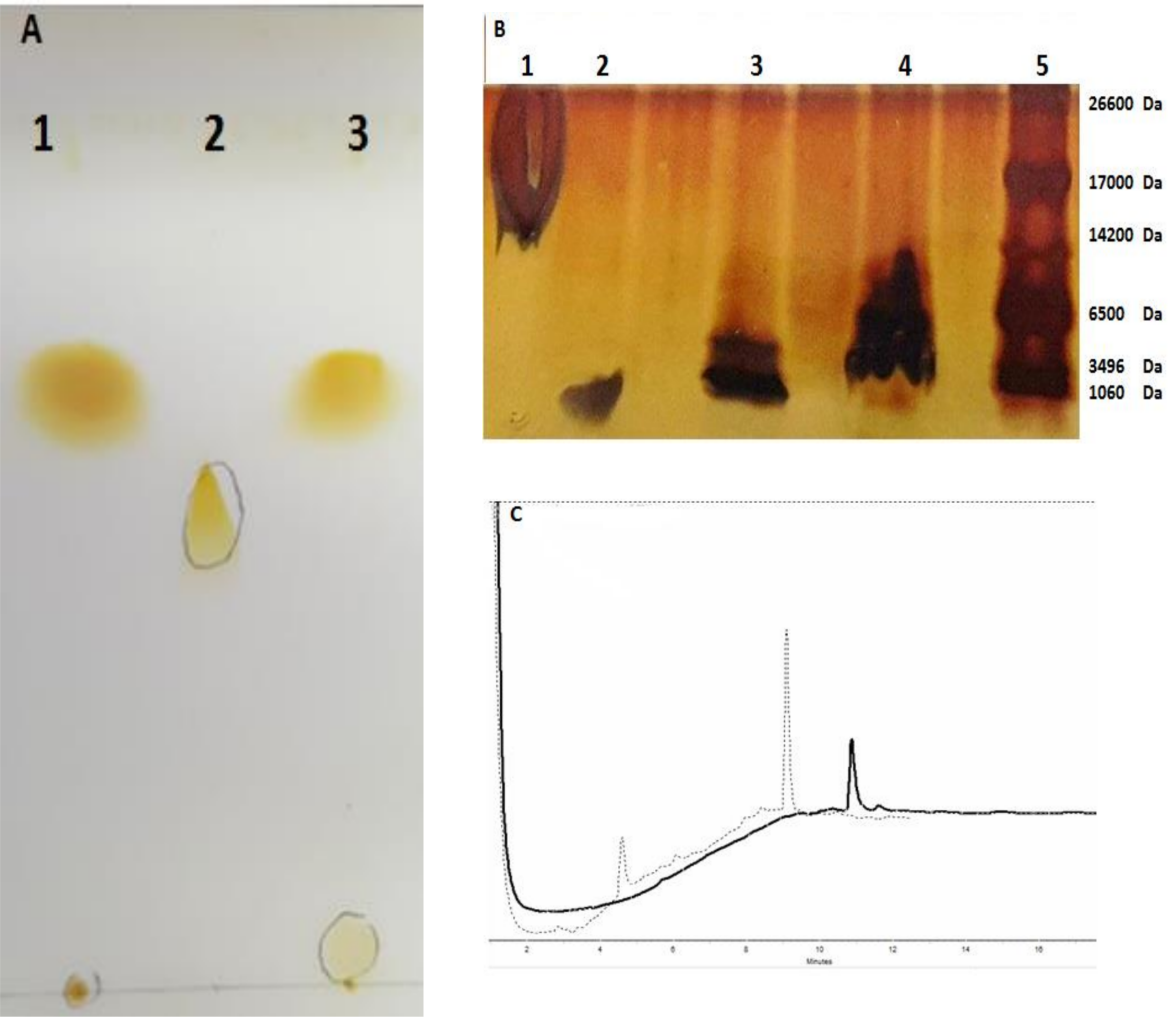
(A) **Thin layer chromatography to show the conjugation of GP2 peptide to Maleimide-PEG-DSPE** (1: GP2 peptide, 2:PEG_2000_-DSPE, 3: GP2-PEG_2000_-DSPE). (B)SDS-PAGE analysis (1: liposome, 2: GP2, 3: GP2-PEG_2000_-DSPE, 4: Lip-GP2, 5: Ladder).(C) Chromatographic analysis of GP2 peptide and the conjugated peptide-PEG-DSPE, included subset graphs are the HPLC monitoring of free peptide as a reference denoted by (-) and the conjugated peptide was determined post reaction with Maleimide-PEG_2000_-DSP denoted by (…).

Formation of the GP2-PEG_2000_-DSPE through binding between the activated maleimide and thiol group of the GP2 peptide was confirmed by TLC and SDS-PAGE. TLC on silica gel 60 F25A indicated the formation of GP2-PEG_2000_-DSPE by the disappearance of The PEG-DSPE spot from the conjugated GP2 peptide (Fig 1A).

The efficiency of the GP2 peptide conjugation was determined by a band shift with respect to increasing molecular weight, as compared toGP2 peptide in denaturing SDS-PAGE. The SDS-PAGE analysis of Lip- GP2 was performed by post insertion method revealed that GP2 peptide was conjugatedto the liposome (Fig 1B).

To further confirm the peptide conjugation, we performed HPLC analysis; free peptide (GP2) was also analyzed as a reference (elution time: ~11). The total peak area, for the GP2-PEG_2000_-DSPE was about twofold higher than that of the reference GP2 peptide (elution time: ~ 9) (Fig 1C).

### Liposome characterization

Physical characteristics of each formulation including average vesicle size, polydispersity index (PdI) and zeta potential were determined and reported in Table 1. All formulations were negatively charged and the size of liposomes ranged from 120 to160 nm which is desirable for a vaccine delivery system. PdI for all formulations was less than 0.2 which indicates a homogeneous population of liposomes [33]. The physicochemical characteristics of liposomes have a significant role in the immune response. The particle size may influence the draining as smaller-sized liposomes have been shown to be cleared faster from the site of injection than larger-sized liposomes [34, 35]. In addition to the size, other factors such as lipid composition impact on the immunity response[35]. The doses of lipid, MPL adjuvant and peptide have a clear impact on the T cell response and efficacy of formulations [16]. According to the lipid dose (4 μm), MPL and peptide doses per mouse were precisely determined as 24 μg and 10 μg, respectively for each formulation.

**Table 1:** Characteristics of liposomal formulations (n = 3; Mean ±SD).

Formulation	Z-average(nm)	Z potential(mv)	PdI^a^
Lip^b^–GP2	143 ± 31	-48 ± 9	0.17± 0.07
Lip-DOPE	135 ± 3	-39 ± 1	0.12 ± 0.02
Lip-DOPE- GP2	130 ± 3	-37 ± 7	0.07 ± 0.04
Lip-MPL-GP2	162 ± 10	-30 ± 1	0.19 ± 0.03
Lip-DOPE-MPL	122 ± 1	-42 ± 1	0.07 ± 0.01
Lip-DOPE-MPL-GP2	130 ± 1	-38 ± 1	0.05 ± 0.01

^a^ Polydispersity index

^b^DMPC-DMPG-Chol

### Induction of CTL response by Lip-DOPE-MPL-GP2 formulation

To determine immune response induced in the immunized mice, INF-γ and IL-4 cytokines production in CD4^+^ and CD8^+^ cells isolated from splenocytes and lymph nodes were measured using flow cytometry. Splenocytes were harvested from immunized mice 14 days after the last immunization. Results demonstrated that the mice immunized with Lip-DOPE-MPL-GP2 produced higher amounts of INF-γ in CD8 population and also indicated greater CTL population compared to the HEPS-dextrose 5% group (*p*<0.001) and Lip-DOPE-MPL(*p*<0.05), and had greater CTL population compared with Lip-DOPE and Lip-GP2 groups (*p*<0. 01).

**Fig 2:**
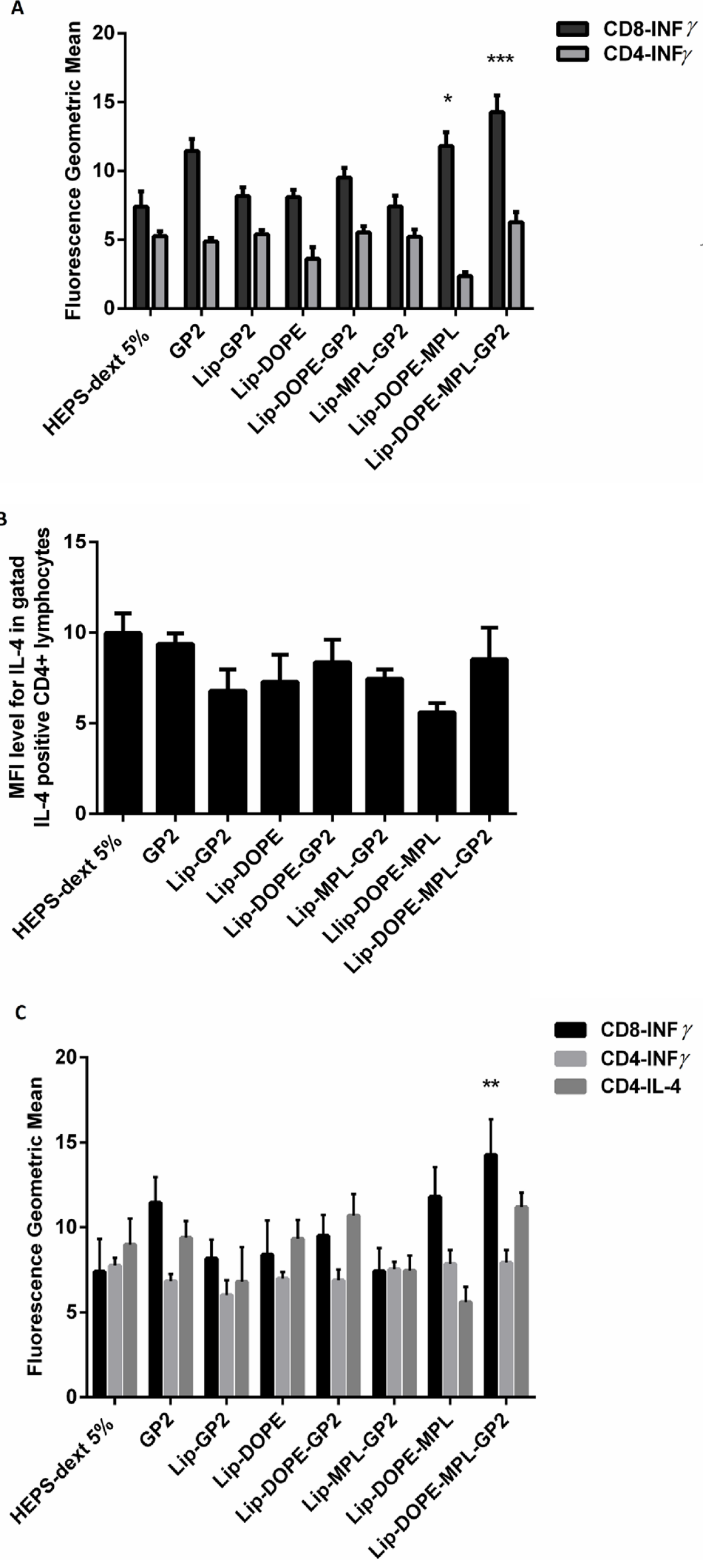
**Splenocyte cell phenotype and level of cytokine expression (A and B) and lymph nodes (C) of BALB / c mice immunized with different liposomal formulations.** 14 days after the last immunization, splenocytes were isolated and stimulated *in vitro* with PMA/I for 4h and stained with a surface CD8 and CD4 marker and intracellular IFN-γ and IL-4 cytokine prior to FACS analysis. (A)Geometric mean fluorescence intensity (MFI) level for INF-γ in gated CD8 and CD4 in the spleen. (B)MFI level for IL-4 in gated CD4 lymphocyte populations in the spleen. (C) MFI level for INF-γ in gated CD8, CD4, and IL-4 in gated CD4 in lymph nodes. Data represent mean± SEM (= 3).*p<0.05, **p<0.01, and ***p<0.001; denote significant difference from all other formulations and the HEPS-dextrose 5%.

This indicates that vaccination with the combination of GP2, MPL, and DOPE induced a significant CTL immune response (Fig 2A).

Based on the MFI of the cells, the CD4 lymphocytes did not produce INF-γ (Fig 2A) and IL-4 (Fig 2B).

The tests on harvested lymph nodes showed that the mice immunized with Lip-DOPE-MPL-GP2 induced higher INF-γ in CD8+ cells compared to the HEPS-dextrose 5% group (*p*<0.01). However, production of INF-γ and IL-4 in CD4 cells was not elicited significantly in all groups (Fig 2C).

### High level of INF-γ in Lip-DOPE-MPL-GP2 formulation

**Fig 3:**
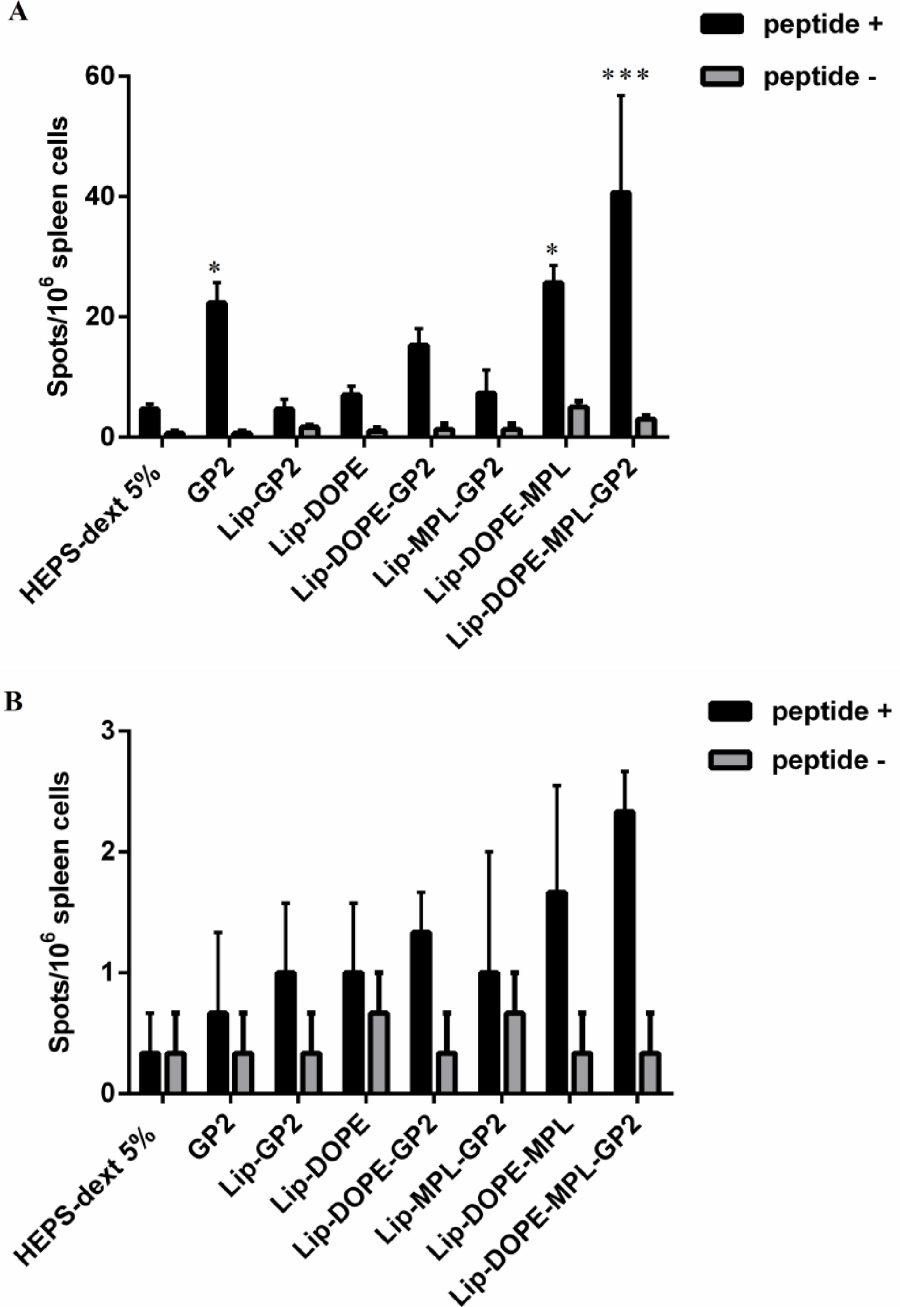
Induction of rHER2/neu peptide-specific intracellular cytokine response in splenocytes as determined by the ELISpot assay. Mice were immunized with three booster doses of 10 μg/mice using different liposomal formulations. Two weeks after the last injection, splenocytes from three mice from each group were harvested and re-stimulated withGP2 peptide (A). Immune responses were evaluated with IFN-γ ELISpot assay kit and (B) IL-4 ELISpot assay kit. The data indicate the mean ±SEM(n = 3).*p<0.05, and ***p<0.001; denote significant difference from all other formulations and the HEPS-dextrose 5%.

To determine the induction of antitumor T cell response, we harvested the splenocytes, 14 days after the last booster. The splenocytes were then stimulated with the GP2 peptide. ELISpot assay showed that splenocytes isolated from the mice immunized with Lip-POPE-MPL-GP2 released higher amounts of INF-γ in comparison to other groups (*p*<0.001). GP2 and Lip-DOPE-MPL vaccination groups could promote the induction of INF-γ against tumor compared to other groups (*p*<0.05) (Fig 3A). As shown in (Fig 3B) none of the formulations induced the sizable IL-4 response in mice.

### Antigen-specific cytotoxicity of Lip-DOPE-MPL-GP2 and GP2

Cytotoxicity assays provide an *in vitro* evaluation of the lytic activity of T cells against tumors or transformed cells [36].

**Fig 4:**
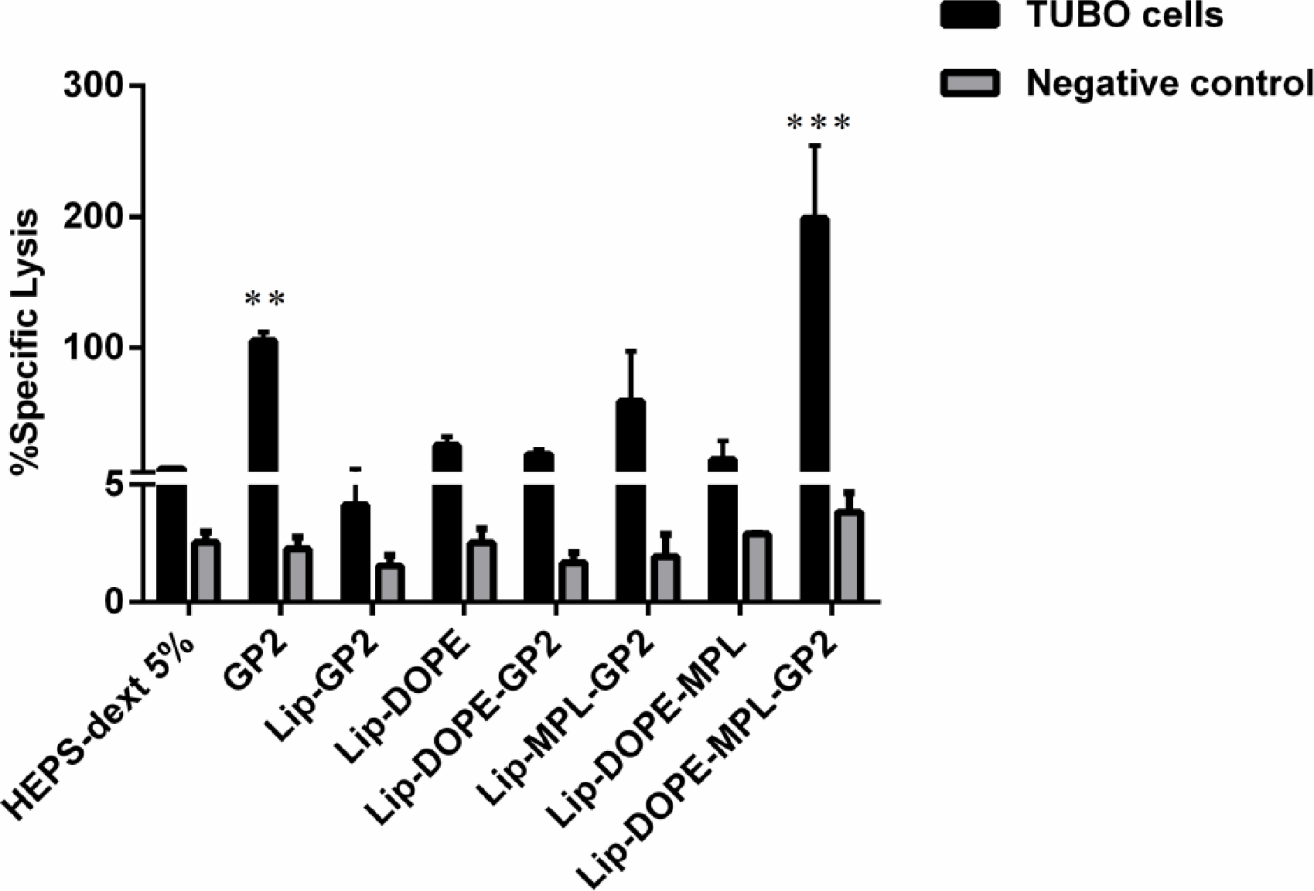
*In vitro* antigen-specific CTL response in different formulations for splenocytes isolated from vaccinated mice. CTL response was assessed by Calcein AM-loadedrHER2/neu-expressing TUBO cells and rHER2/neu negative CT26 cells, Data are shown as mean ± SEM (n = 3). **p<0.01, and ***p<0.001; denote significant difference from the HEPS-dextrose 5%.

Lip-DOPE-MPL-GP2 and GP2 liposomal formulations were significantly effective at generating CTL response and reacted with the TUBO cell line expressing rHER2/neu in comparison with the HEPS-dextrose 5%group (*p*<0.001, *p*<0.01). This response was antigen specific because the CTL response was not observed against CT26 tumor cells that are rHER2/neu-expressing negative (Fig 4).

### Expression of IFN-γ and IL-4

**Fig 5:**
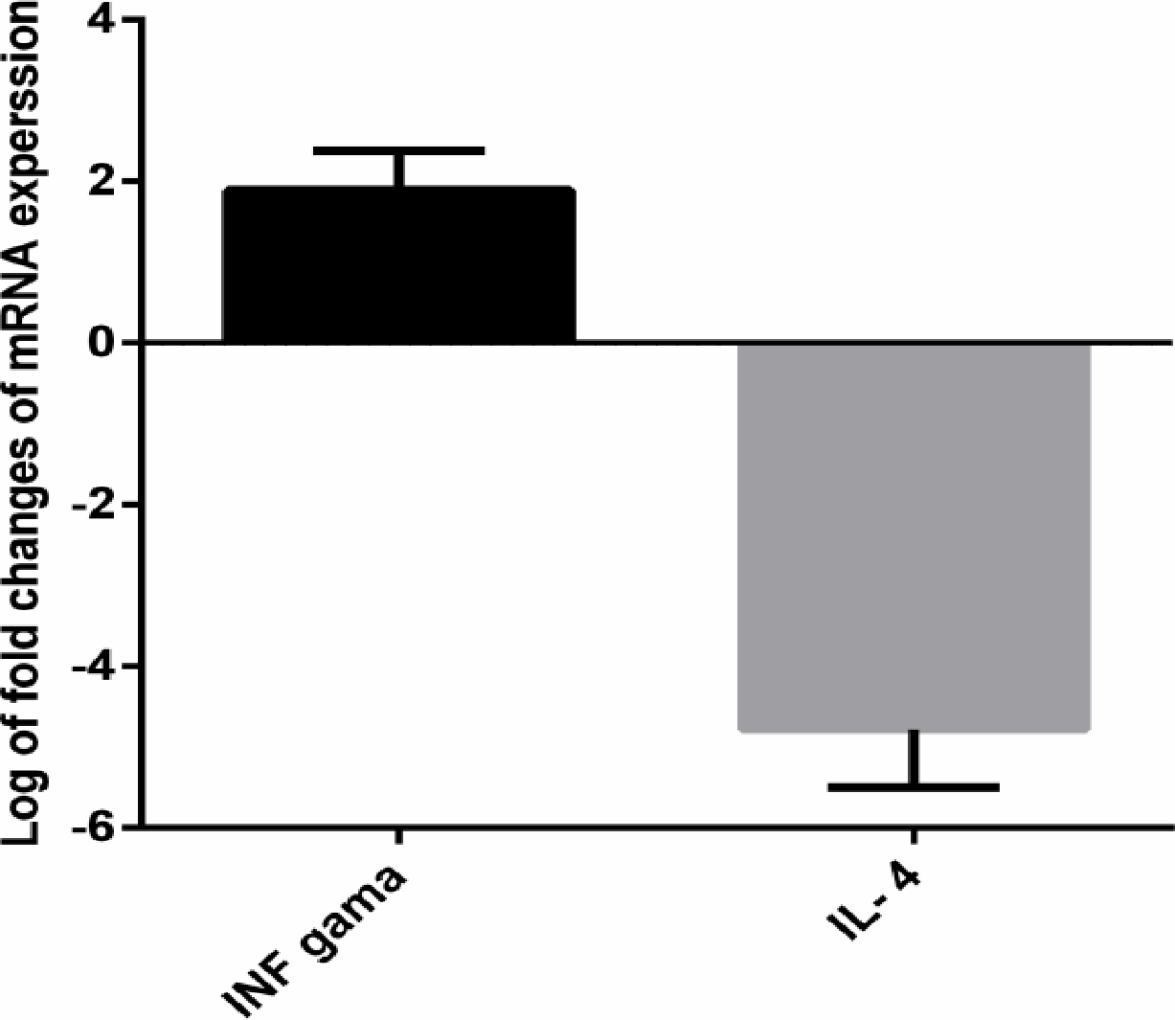
qRT-RCR. Analysis of IFN-γ and IL-4 levels in splenocytes isolated from BALB/c mice vaccinated with Lip-DOPE-MPL-GP2, two weeks after the final vaccination. All values represent means ± SD (n = 3).

As mentioned in the above lines, the Lip-DOPE-MPL-GP2 formulation showed the highest levels of CTL response in the immunized mice compared to the other formulations. This was supported by real time RT-PCR analysis indicating that Lip-DOPE-MPL-GP2 formulation modulated mRNA expression of both IFN-γ and IL-4 cytokines in favor of CTL immune response effectively. The results demonstrated that IFN-γ was increased by 1.85 ±0.4 (95% CI: 0.35, 3.33 *р*<0.001) in splenocytes of mice immunized with Lip-DOPE-MPL-GP2 compared to the HEPS-dextrose 5% group 14 days after the last immunization, whereas IL-4 expression was found to be significantly decreased by 4.26819±1.5 (95% CI: -5.2, -3.2, *р*<0.001)(Fig 5).

### Prophylactic assays

**Fig 6:**
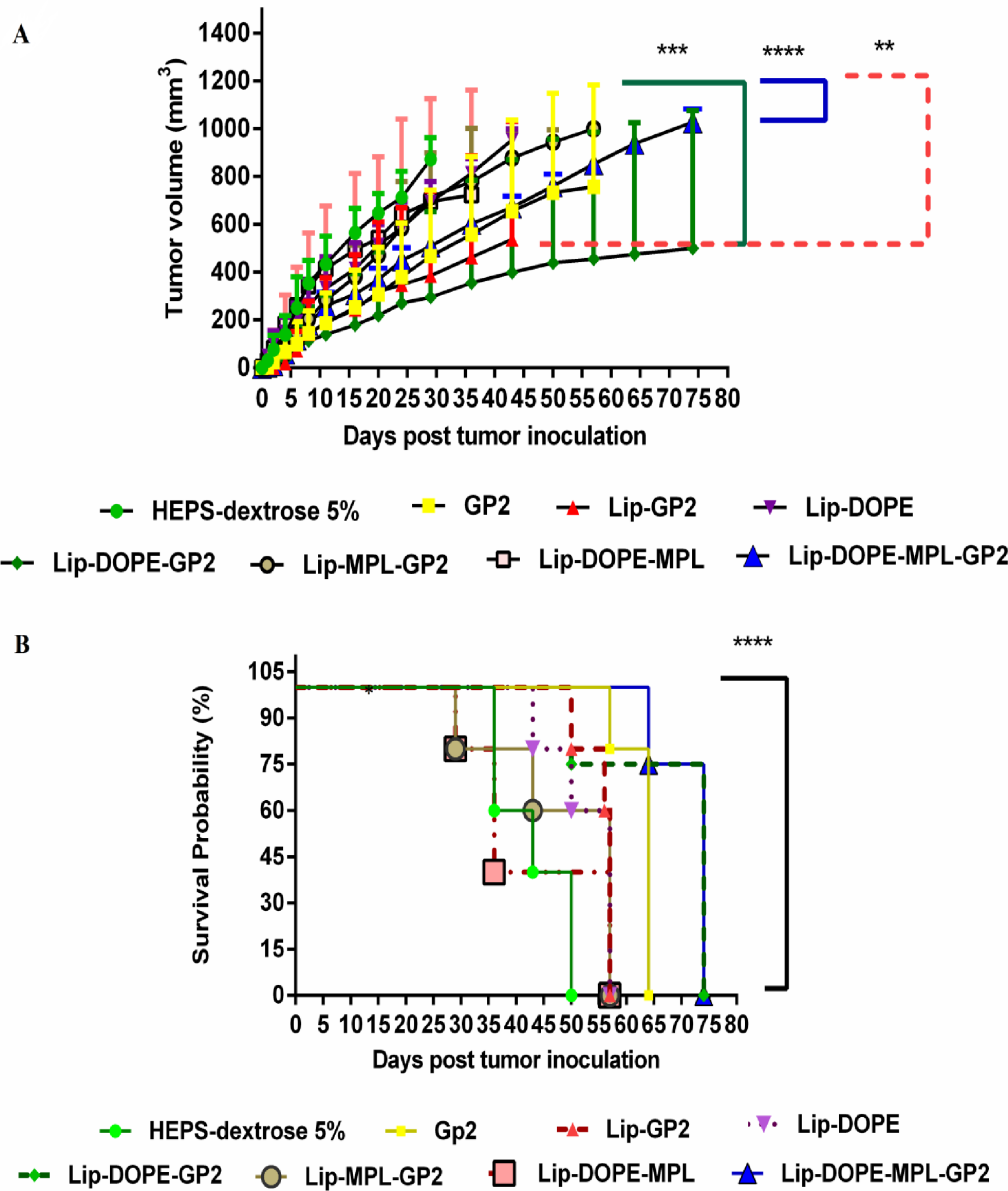
Prophylactic effects of vaccination in BALB/c mice against a TUBO tumor model. Two weeks after the last booster, five mice in each group were challenged subcutaneously with 5 ×10^5^ TUBO cells. Mice were observed for tumor growth (A) and survival (B). Tumor size was calculated based on three dimensions, two times every week. The survival of mice was followed for about 75 days. The data indicate the mean± SEM (n = 5). **p<0.01, ***p<0.001, and **** *p*<0.0001; denote significant difference from the HEPS-dextrose 5% group.

Tumor growth curve analysis indicated that Lip-DOPE-MPL-GP2 (*p*<0.0001) and Lip-DOPE-GP2 (*p*<0.001) groups were the most effective formulations in terms of reducing the growth rate of the tumor (Fig 6A). The prophylactic effect of the liposomal formulations in the mouse model is summarized in Table 2, including median survival time (MST), time to reach end point (TTE), and tumor growth delay (% TGD) for each mouse group.

**Table 2:** Prophylactic efficacy data of different liposomal vaccine formulations in TUBO tumor model of mice (n = 5).

Formulation	MST ^a^ (day)	TTE ^b^(day ± SD)	TGD ^c^%
HEPS-dextrose 5%	43	39 ± 7.3	-
GP2 peptide	64	61 ± 6.7	58
Lip-GP2	57	58 ± 5.8	48
Lip-DOPE	57	48 ± 3.3	23
Lip-DOPE-GP2	74	70.± 12.2	81
Lip-MPL-GP2	57	45 ± 13.2	15
Lip-DOPE-MPL	36	40 ± 16.0	4
Lip-DOPE-MPL-GP2	74	75 ± 7.2^"****"^	93

^a^Median survival time.

^b^Time to reach end point.

^c^Tumor growth delay.

^"****"^Denotes significant difference from all other formulations.

Survival analysis (up to 70 days) revealed that Lip-DOPE-MPL-GP2 and Lip-DOPE-GP2 groups had a higher protective effect compared to all other formulations. These data suggest that prophylactic effect of Lip-DOPE-MPL-GP2 and Lip-DOPE-GP2 groups were %TGD of 92.22% and 80.88%, respectively. This plays a critical role in long-term survival (Fig 6B).

### Therapeutic assays

**Fig 7:**
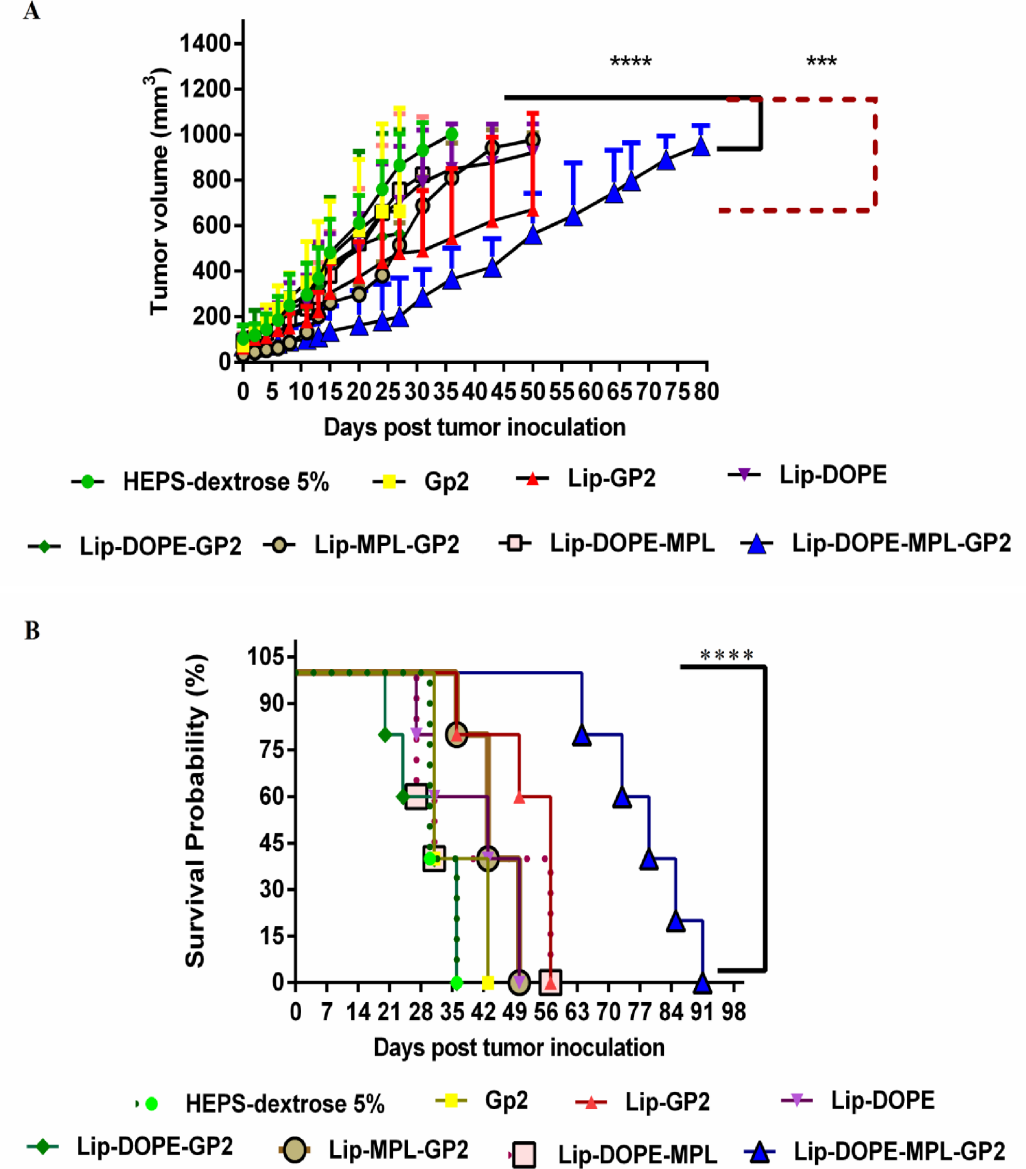
Therapeutic effects of liposomal formulations in BALB/c mice against a TUBO tumor model. Two weeks after inoculation of 5 ×10^5^ TUBO cells to five mice in each group, different liposomal formulations were administrated three times with two-week intervals. After the first injection, the mice were challenged and tumor size was calculated based on three dimensions. (A) Tumor growth was measured two times every week. (B) The survival of mice was followed for 91 days. The data indicate the mean ±SEM(n = 5). ***p<0.001 and ****p<0.0001; denote significant difference from the HEPS-dextrose 5% group.

Following the considerable T cell responses in immunized mice, anti-tumor activity in TUBO tumor model was evaluated. Among different formulations, Lip-DOPE-MPL-GP2 was superior in inhibition of tumor growth rate compared to the HEPS-dextrose 5% group (*p*<0.0001) (Fig 7A). The therapeutic efficacy of liposomal formulations in the mouse model is summarized in Table 3 including median survival time (MST), time to reach end point (TTE), and tumor growth delay (%TGD) for each mouse group.

**Table 3:** Therapeutic efficacy data for liposomal formulations in TUBO tumor mice model (n = 5).

Formulation	MST^a^ (day)	TTE ^b^(day±SD)	TGD ^c^ %
HEPS-dextrose 5%	30	33 ± 4.1	-
GP2 peptide	31	35 ± 7.7	6
Lip-GP2	57	52 ± 11.7	55
Lip-DOPE	43	48 ± 21.7	43
Lip-DOPE-GP2	31	35 ± 11.5	5
Lip-MPL-GP2	43	45 ± 12.9	34
Lip-DOPE-MPL	31	37 ± 13.8	13
Lip-DOPE-MPL-GP2	79	80 ± 12^"****"^	144

a Median survival time.

b Time to reach the end point.

c Tumor growth delay.

^"****"^Denotes significant difference from all other formulations.

The survival analysis results represented in a Kaplan-Meier plot were used to analyze significant differences in therapeutic efficacy between different treatment groups. Survival data showed that mice treated with Lip-DOPE-MPL-GP2 liposomal formulation had the longest MST in comparison to all other formulations. Lip-DOPE-MPL-GP2 with 143.83%TGD,showed the highest anti-tumor efficacy and revealed extended survival time relative to the other formulations (Fig 7B).

## Discussion

The aim of this study was to generate a cancer vaccine based on immunogenic GP2 peptide with the effective adjuvant using a suitable delivery system. We prepared different liposomal formulations with GP2 peptide to induce enhanced CTL immune response in a TUBO tumor model of mice.

GP2 peptide as a TAA has broad applicability as a cancer immunogen with low toxicity[9]. In a phase II clinical trial using granulocyte–macrophage colony-stimulating factor (GM-CSF) as a vaccine adjuvant, it has been reported that GP2 decreased the risk of recurrence in women with HER2/neu breast cancer[37]. In another study, Brossart and Dess reported that injection of GP2 and GP2-pulsed dendritic cells against metastatic breast cancer and advanced ovarian cancer patients induced CTL immune response[11, 38]. It has also been shown that the GP2 based vaccines are effective in stimulating peptide-specific immunity, especially in CD8+ T cell stimulation with anti-tumor activity in human leukocyte antigen (HLA)-A2 + breast cancer patients[37]. The GP2 peptide had immunogenicity in HER2/neu positive breast cancer and could be used in a multi epitope vaccine formulation [39]. Recent studies revealed that patientsvaccinatedwithGP2+GM-CSF showed a 37% reduction in cancer recurrence compared to unvaccinated patients while those who received GM-CSF alone showed57% reduction in risk of cancer recurrence [40]. However, Mittendorf reported that local and systemic toxicity is due to the GM-CSF use, in most patients usually experienced as grade1 headache, fatigue, erythema, pruritus, bone pain, myalgia, and flu-like symptoms [12]. Of course, GM-CSF can induce antitumor immune response but it promotes cancer cell proliferation and migration in different solid tumors such as skin carcinoma, gliomas, lung cancer, and cancer cell line[41–43]. It has been shown that pretreatment of three tumor cell lines of breast cancer with trastuzumab followed by incubation with GP2 peptide induced CTL immune response[44]. The immunogenic potency of the GP2 peptide for applying in HER2-positive breast cancer therapy in combination with other peptides or with the monoclonal antibody trastuzumab has been investigated [39]. The GP2+GM-CSF primary vaccine series (PVS) consisted of six inoculations and boosters given every 21–28 days [12], which involves a time and cost consuming procedure.

Liposomes as carriers of vaccines and drugs are safe [45]. Liposome vaccine delivery provides more efficacy and safer vaccine formulation in the development of a vaccine for human use[46]. Liposomes have a major advantage as an antigen delivery system since they have a long circulation time and tendency to be taken up more efficiently by APCs to induce CTL response [47, 48]. The challenge of inducing strong CTL response could be potentially overcome by using the liposomal adjuvant/ delivery systems. Liposomes can reclaim peptide antigen delivery and elevate cellular uptake with dendritic cells[18, 49]. The current study demonstrates for the first time, the significant benefit of GP2 peptide conjugation toliposomes with MPL to induce CD_8_+ T cell response. The use of MPL in liposomes with aHER2/neu peptide has been reported before [24]. This study aimed at stimulation of CTL response by GP2HER2/neu peptide and liposomes, hoping that this may be a major substantial approach, considering the cost of manufacturing recombinants and side effects of treatment with GM-CSF.

Various animal studies have shown that liposomal formulations have higher antitumor efficacies compared to non-liposomal vaccine systems [50–52].Liposomal vaccines with basic fibroblast growth factor(bFGF) and MPL adjuvant induced Th1 and tumor-specific antibody response immunity in a mouse model[19]. In addition, liposomes as immune adjuvants could provoke cellular and humoral immunity against the antigen [53, 54]. E7 is a peptide antigen which is derived from E7 oncoprotein of human papillomavirus (HPV) type 16. The DOTAP (the cationic lipid) /E7 formulation induced both preventative and therapeutic antitumor effects against HPV positive TC-1 tumor in a mouse model[55]. Also, the conjugation of the MUC1 peptide (tumor-associated antigen in lung cancer) to the surface of a liposome with a composition of DPP-DPPG-Chol (3:1:0.25, molar ratio) containing MPL A (1% w/w of the total lipids) elicited strong CTL response[56]. Both Tc-ErbB2/Th-HA peptide antigens, conjugated to the surface of liposomes via aPam3CSSanchor, can induce potent antigen-specific immune responses in the majority of tumor-bearing mice and delay tumor growth in BALB/c mice model [57]. A recent study suggested that the covalently linked conalbumin antigen to DMPC-DPPE-Chol liposom stimulates more secretion of IL-2 and 1FNγ compared to the encapsulated antigen. So covalently linked antigen might be particularly useful, its induction of cell-mediated immunity is of prime importance[23].

In this study, we used GP2 peptide conjugated to the DOPE-based pH-sensitive liposomes in the presence of MPL as an adjuvant for the anti-cancer vaccine. MPL induces the development of Th1 response and can be connected and activated to the Toll-like receptor4 (TLR4), which plays an important role in the induction of immune response[58]. MPL as a cancer vaccine adjuvant has been tested in several clinical trials [58]. Reportedly, MPL is safe and immunogenic in human clinical trials [58, 59]and can efficiently deliver the peptide into the APCs to increase the elicitation of antigen-specific immune response[51, 60]. Zollinger reported that *N*. *meningitides* liposomal vaccine was safe in healthy adults and induced bactericidal antibodies[61].

DOPE as a fusogenic lipid can increase the efficiency of liposomal vaccine delivery [22, 62]. The liposome containing DOPE is stable at physiological pH but destabilizes upon acidification (pH<6.5) following cellular internalization through the phase transition DOPE from lamellar to hexagonal.[63]. It has been shown that DOPE-based pH-sensitive liposomes improve the cytoplasmic delivery of biological membrane-impermeable therapeutic agents [64, 65].Our results indicated that free GP2 did not have a significant therapeutic effect in mice and exhibited a low prophylactic effect in terms of tumor growth inhibition. Due to rapid distribution of proteins or synthetic peptides to other organs from the site of injection, antigen specific CTL would fail in a short time[47, 66]. Despite the suitable size of Lip-GP2 (142.5 nm) for antigen presentation to lymph nodes [33], this formulation could not stimulate a sufficient CTL response. Addition of MPL and DOPE to the liposome (Lip-DOPE-MPL), increased stimulation of CD8^+^ cells, and production of IFNγ in mice splenocyte was enhanced. However, the Lip-DOPE-MPL liposome could not inhibit tumor growth. Due to the absence of GP2 antigen in Lip-DOPE-MPL, this formulation would not induce considerable CTL response and long term protective immunity against her2/neu tumor in mice [67]. Although the Lip-DOPE formulation could not induce enough immune response, inclusion of the GP2 (Lip-DOPE-GP2) was able to deliver GP2 to MHCI and activate CD8 cells to produce CTLs. Survival tests indicated that vaccination with Lip-DOPE-GP2 had a significant protective effect (*p*<0.001) compared to other groups. It should be noted that Lip-DOPE-GP2 did not show effectiveness in the therapeutic assay and also Lip-MPL-GP2 was unable to induce any immune response. These observations indicate the crucial role of MPL and DOPE in the formulation. Inclusion of DOPE could destabilize the liposome-Gp2 binding in lysosomes, facilitating the release of GP2 from the liposome to the MHC I molecule and therefore enhancing an antigen-specific CTL response[68]. Vaccination with Lip-DOPE-MPL-GP2 resulted in significant CTL response in mice and provided the highest level of the significant protective effect (*p*<0.0001). It also exhibited a potent therapeutic effect (*p*<0.0001) compared to HEPS-dextrose 5% group. The CTL response was found to be associated with higher and lower amounts of IFN-γ and IL-4, respectively, in both protein and mRNA expression studies. The mice vaccinated with Lip-DOPE-MPL-GP2 showed a delay in tumor growth and had a longer survival time in prophylactic and therapeutic assays.

All in all, our findings showed that the presence of co-stimulator molecules such as MPL and DOPE with antigenic properties of GP2 enhanced the ability of APCs to induce the IFN-γ producing CD8^+^ cells and increased the level of IFN-γ as a major anti-tumor and Th1 type immunity cytokine.

## Conclusion

We demonstrated that the GP2 peptide conjugation on the surface of a liposome composed of DMPC- DMPG- Chol-DPOE and MPL adjuvant increased splenocytes INF-γ production, which can be applied prophylactically as well as therapeutically to reduce tumor growth in HER2/neu-overexpressing TUBO breast cancer model. This formulation can be a potential candidate for developing a liposomal vaccine as a protective and inhibitor of tumor in HER2/neu breast cancer and merits further investigation.
